# Fluorescent Nanodiamonds Enable Long-Term Detection of Human Adipose-Derived Stem/Stromal Cells in an In Vivo Chondrogenesis Model Using Decellularized Extracellular Matrices and Fibrin Glue Polymer

**DOI:** 10.3390/polym11091391

**Published:** 2019-08-23

**Authors:** Yi-Chia Wu, Ya-Chin Wang, Wei-Ting Wang, Hui-Min David Wang, Hsin-Hung Lin, Long-Jyun Su, Yur-Ren Kuo, Chung-Sheng Lai, Mei-Ling Ho, John Yu

**Affiliations:** 1 Institute of Stem Cell and Translational Cancer Research, Chang Gung Memorial Hospital at Linko, Taoyuan 333, Taiwan; 2 Ph.D. Program in Translational Medicine, Kaohsiung Medical University, Kaohsiung, and Academia Sinica, Taipei 115, Taiwan; 3 Regeneration Medicine and Cell Therapy Research Center, Kaohsiung Medical University, Kaohsiung 807 Taiwan; 4 Division of Plastic Surgery, Department of Surgery, Kaohsiung Medical University Hospital, Kaohsiung 807, Taiwan; 5 Center of Teaching and Research, Kaohsiung Municipal Ta-Tung Hospital, Kaohsiung 801, Taiwan; 6 Graduate Institute of Biomedical Engineering, National Chung Hsing University, Taichung 402, Taiwan; 7 Institute of Atomic and Molecular Sciences, Academia Sinica, Taipei 106, Taiwan; 8 Orthopaedic Research Center, Kaohsiung Medical University, Kaohsiung 801, Taiwan; 9 Institute of Cellular and Organismic Biology, Academia Sinica, Taipei 115, Taiwan

**Keywords:** human adipose-derived stem/stromal cells (hASCs), in vivo chondrogenesis, decellularized cartilage ECM, fibrin glue polymer, fluorescent nanodiamonds (FNDs), long-term detection

## Abstract

Clinically available materials, including allogeneic irradiated costal cartilage and fibrin glue polymer, were used as scaffolds for in vivo chondrogenic differentiation of human adipose-derived stem/stromal cells (hASCs) in the attempt to develop a more efficient treatment over current methods. Current studies include the use of growth-factor stimulation, tissue engineering, and biocompatible materials; however, most methods involve complicated processes and pose clinical limitations. In this report, the xenografts in the experimental group composed of a diced decellularized cartilage extracellular matrix (ECM), hASCs, and fibrin glue polymer were implanted into the subcutaneous layer of nude mice, and the results were compared with two groups of controls; one control group received implantation of decellularized cartilage ECM and fibrin glue polymer, and the other control group received implantation of hASCs mixed with fibrin glue polymer. To evaluate whether hASCs had in vivo chondrogenesis in the xenografts, hASCs were labeled with fluorescent nanodiamonds (FNDs), a biocompatible and photostable nanomaterial, to allow for long-term detection and histological analysis. Increased cellularity, glycosaminoglycan, and collagen deposition were found by the histological examination in the experimental group compared with control groups. With the background-free detection technique and time-gated fluorescence imaging, the numbers and locations of the FND-labeled hASCs could be detected by confocal microscopy. The chondrocyte-specific markers, such as aggrecan and type II collagen, were colocalized with cells containing signals of FNDs which indicated in vivo chondrogenesis of hASCs. Taken together, functional in vivo chondrogenesis of the hASCs could be achieved by clinically available decellularized cartilage ECM and fibrin glue polymer in the nude mice model without in vitro chondrogenic induction. The fluorescent signals of FNDs in hASCs can be detected in histological analysis, such as hematoxylin and eosin staining (H&E staining) without the interference of the autofluorescence. Our study may warrant future clinical applications of the combination of decellular cartilage ECM, fibrin glue polymer, and hASCs for cartilage repair.

## 1. Introduction

Articular cartilage is a highly specialized tissue acting as an absorber of weight during sustained static loading that cushions the ends of bones where they come together to form joints [1]. As an avascular tissue with low cellular biosynthetic activity, cartilage has limited repair potential [2,3]. Articular cartilage damage from trauma or degenerative pathology often results in progressive deterioration, leading to joint pain, functional impairment, and degenerative arthritis [1,2]. Surgical interventions, aiming to either replace damaged chondral tissue or to induce repair, include arthroscopic debridement [4], bone marrow stimulation techniques [5,6], chondrocyte implantation [7,8], osteochondral autografts [9], osteoarthritis allograft [10], and total joint replacement for end-stage degenerative joint pathology [11].

Many of these approaches, however, often result in the formation of fibrocartilage that is biochemically and biomechanically inferior to hyaline articular cartilage. Autologous chondrocyte implantation and its later relevant approach, matrix-induced autologous chondrocyte implantation, offer great promise in clinical and histological results [12,13], but many adverse complications have been reported, including graft failure, delamination, and tissue hypertrophy [14,15]. In addition, the ability of autologous chondrocytes to regenerate cartilage neotissues decreases with patients’ age and in vitro expansion [16], making them a substandard cell source for cartilage repair. The repair of cartilaginous lesions is still a weighty clinical problem.

Development of stem-cell-based therapies is one of the most promising advancements in cell therapy and regenerative medicine. Mesenchymal stem/stromal cells (MSCs) derived from tissues such as bone marrow, adipose, or placenta are defined as self-renewal, multipotent progenitor cells with the ability to differentiate into multiple lineages, such as adipocytes, chondrocytes, and osteocytes [17]. MSCs are one of the ideal sources for stem-cell-based therapies due to their multilineage differentiation potential and immunomodulatory properties [18]. hASC is an ideal source for cartilage-tissue engineering, owing to their accessibility, capacity for self-renewal, and potential to synthesize cartilage-specific matrix proteins that are assembled in a cartilage ECM [19,20].

Various studies have reported that tissue engineering of cartilage has been targeting on scaffolds, cells, and addition of growth factors [21,22]. Allogenic costal cartilage may be used as a decellularized cartilage ECM for the surgical reconstruction of damaged cartilaginous tissue. The combination of decellularized cartilage ECM, hASCs, and fibrin glue polymer for cartilage regeneration could solve several shortcomings, such as donor site morbidity, limited cell sources, and multiple surgeries [3]. In addition, fibrin has been widely applied as a scaffold in clinical purposes and is one of the suitable scaffolds for chondrogenic differentiation of stem/stromal cells [23,24]. Fibrin scaffold provides a useful platform and appropriate environment for chondrogenesis of hASCs [25] because of its adhesive properties, biocompatibility, and biodegradation [26]. TISSEEL is mainly composed of fibrinogen (85 mg/mL) and thrombin (500 IU/mL), which are mixed in a 1:1 ratio in the course of application. The main protein components of finial fibrin glue polymers are fibrin, fibronectin, and albumin. The fibrin concentrations within the clots are 45 mg/mL. It polymerizes on its own and was approved by the U.S. Food and Drug Administration for hemostats, sealants, and adhesives [27,28]. The hASCs were adhesive to the decellularized cartilage ECM to form the xenograft constructs in our experiments. Taken together, a fibrin scaffold composed of clinically available decellularized cartilage ECM and fibrin glue polymer could be an ideal material for in vivo chondrogenesis of hASCs.

Accurate stem-cell tracking in vivo is one of the most important requirements in regenerative medicine. Cell tracking determines both stem cell destinations and final differentiation fates, thus allowing a more detailed picture of the mechanisms involved in these therapies [21]. In order to investigate the efficacies and safety throughout the course of stem-cell-based treatment, it is vital to develop a method for tracking the stem cells. [22]. At present, long-term tracking of cells is one of the most difficult hurdles to overcome for studying in vivo chondrogenesis. Fluorescent nanodiamonds (FNDs) [29] could be an ideal long-term cell tracking device for studying in vivo chondrogenesis. FND is a carbon-based nanomaterial [30] with extraordinary chemical stability and biological inertness [29,30]. A number of studies have demonstrated that FNDs can be internalized by cells through clathrin-mediated endocytosis [31] with low exocytosis activity [32,33]. FNDs can be widely used for biomedical research as a novel nanotechnology for image probing [34,35]. The negatively charged nitrogen-vacancy (NV^−^) color centers in the diamond lattice of FNDs as built-in fluorophores which have perfect photostability, without photobleaching and blinking [29]. When the greenish-yellow light excites the NV^−^ centers of FNDs by a laser, the NV^−^ centers emits stable far-red emission (around 600–800 nm) with the fluorescence lifetime (τ) being longer than 15 ns, which can avoid the interference of a strong autofluorescence background from the host tissue using time-gating techniques [32]. Taking advantage of the unique magneto-optical property of NV^−^ centers, the absolute number of transplanted cells can be quantified by magnetically modulated fluorescence (MMF), a background-free detection method [34].

In this work, hASCs labeled with FNDs had unaffected cellular functions, such as proliferation and differentiation. The xenografts of the experimental group composed of the decellularized cartilage ECM, fibrin glue polymer, and hASCs were implanted into nude mice. FND-labeled hASCs were clearly identified and quantified in xenografts three months afterwards. The xenografts of the experimental group had increased cellularity and volume compared with the control groups. Furthermore, increased glycosaminoglycans and collagen were also observed. The chondrocyte-specific markers, such as aggrecan and type II collagen, were colocalized with the signals of FNDs, which might indicate in vivo chondrogenesis of hASCs. The FNDs are applicable for long-term detection of stem/stromal cells which might undergo cell differentiation.

## 2. Materials and Methods

### 2.1. FNDs Production and Characterization

Synthetic type Ib diamond powders (Micron + MDA, Element Six, London, UK) with a mean size in the range of ~100 nm were radiation-damaged by ion bombardment with 40-keV He+, followed by annealing in vacuum at 800 °C, oxidization in air at 490 °C, and purification in concentrated H_2_SO_4_–HNO_3_ (3:1, v/v) at 100 °C, as previously described [35]. The size and spectrum of FNDs were determined by Fluorescence spectrometer and dynamic light scattering. Normalized fluorescence spectrum of FNDs and human serum albumin (HSA)-conjugated FNDs (FND@HSA) in water (1 mg/mL) were acquired by laser excitation at 532 nm. Size distributions of the particles in both distilled deionized water and PBS were measured with a combined particle size and zeta potential analyzer (Delsa Nano C, Beckman-Coulter, Brea, CA, USA).

### 2.2. Culture of Human Adipose-Derived Stem/Stromal Cells (hASCs)

The hASCs were purchased from Lonza (PT-5006, Bazel, Switzerland) and cultured with complete K-medium [Defined Keratinocyte-SFM (Thermo Fisher Scientific, Waltham, MA, USA) supplemented with 5% FBS (Thermo Fisher Scientific, Waltham, MA, USA), 1% Antibiotic-Antimycotic (Thermo Fisher Scientific, Waltham, MA, USA), 57.8 μg/mL L—Ascorbic acid, 2-phosphate sesquimagnesium salt hydrate (Sigma-Aldrich, St. Louis, MO, USA), and 326.4 μg/mL N—Acetyl-L-cysteine (Sigma-Aldrich, St. Louis, MO, USA) in a humidified incubator at 37 °C with 5% CO_2_.

### 2.3. Cell Labelling

Prior to cell labeling, 100 nm FNDs were first dispersed in serum-free DMEM and then diluted with the medium to have a concentration in the range of 5–200 μg/mL, as indicated. hASCs were plated in culture dishes and labeled with FNDs in serum-free medium at 37 °C with 5% CO_2_ for four hours, and then thoroughly washed with PBS to remove free FNDs by centrifugal separation.

### 2.4. MTT Assay

1 × 10^5^ hASCs were seeded into 6-well culture dishes overnight to allow cell attachment before FNDs treatment. Afterwards, cells were refreshed with medium containing 10 μg/mL MTT under 37 °C, 5% CO_2_ in the dark for two hours. Dimethyl sulfoxide (DMSO) was used to dissolve formazan crystals produced by living cells. Cell viability was determined and quantified by measuring absorbance at a wavelength of OD 570.

### 2.5. Flow Cytometry Analysis

Single-cell suspensions of hASCs were stained. Aliquots of 1 × 10^4^ cells were labeled with fluorescein isothiocyanate (FITC)—conjugated monoclonal antibodies against human CD29, CD31, CD34, CD44, CD45, and CD90 for 30 min at 4 °C. The cells were washed three times in cold PBS and analyzed using a BD LSR II (BD Biosciences, San Jose, CA, USA). Antibodies recognizing CD29, CD31, CD34, CD44, CD45, and CD90 were purchased from Abcam (Cambridge, UK).

### 2.6. In Vitro Multipotency Differentiation Assay

The hASCs, no later than passage three, were plated into 6-well plates at a density of 10^6^ cells/well and treated with 100 μg/mL FNDs. The cells were then induced to adipogenic, osteogenic, or chondrogenic differentiation by using hMSC adipogenic induction SingleQuots™ kit (Lonza PT-4135, Bazel, Switzerland) or a hMSC osteogenic SingleQuots™ kit (Lonza PT-4120, Bazel, Switzerland) or Human Mesenchymal Stem Cell Chondrogenic Differentiation Medium BulletKit^TM^ (Lonza PT-3003, Bazel, Switzerland) following the instruction manual provided with the kit. These differentiated lineages derived from hASCs were further confirmed by Oil Red O (adipogenesis) or Alizarin Red (osteogenesis) or Alcian Blue (chondrogenesis) staining, respectively.

### 2.7. Animals and In Vivo Implantation

Thirty nude male mice purchased from the National Laboratory Animal Center at five weeks of age were acclimatized for up to six weeks prior to use. The animal protocol was followed and approved by the Kaohsiung Medical University Animal Care Advisory Committee (No. 106056). Mice were housed and cared for at the animal facility of Kaohsiung Medical University. The mice were randomly allocated into three groups (ten mice/group): (1) the ECM-glue group, composed of diced decellularized cartilage ECM and fibrin glue polymer (TISSEEL Solution; Baxter AG; Vienna, Austria); (2) the ECM-hASC-glue group, composed of 1 × 10^5^ hASCs in diced decellularized cartilage ECM and fibrin glue; or (3) the hASC-glue group, composed of 1 × 10^5^ hASCs in fibrin glue polymer.

### 2.8. Xenograft Construct Preparation and Measurement

We grounded the allogeneic irradiated costal cartilage (LifeNet, Virginia Beach, VA, USA) into particles approximately 0.5 mm × 0.5 mm × 0.5 mm in size as decellularized cartilage ECM in this study. The grounded cartilages were loaded into a syringe (Figure S3a). The fibrin glue polymers, with or without hASCs, were then loaded into the syringe (Figure S3b). The volume of each xenograft was 0.5 mL. The xenografts were implanted subcutaneously into six-week nude mice for three months. Three months later, the xenografts were retrieved and placed in a syringe, and injected into the syringe with PBS to a volume of 1 mL. The xenograft was taken out, and the volume of the PBS was then recorded. The xenografts were then subjected for histological analysis and MMF.

### 2.9. Histological Analysis

The harvested xenografts were fixed in 4% paraformaldehyde (Sigma, St. Louis, MO, USA) overnight and stored in 70% ethanol at 4 °C for further processing. Xenografts were then embedded in paraffin and processed following the standard histological procedures. The xenograft structures were sliced into sections which were 5 μm thick. Sections were stained with hematoxylin and eosin.

### 2.10. Quantification of Cell Number

The harvested xenografts were sliced into sections which were 5 μm thick. Each section was mounted with fluorescence mounting medium, including 4′,6-diamidino-2-phenylindole (DAPI) (ab104139, Abcam, Cambridge, MA, USA) for counterstaining. The image of the section was taken under a fluorescence microscope with 20-fold magnification. The cell numbers in six random fields of each section were counted.

### 2.11. Alcian Blue Staining

The sections were fixed with 3.7% formaldehyde in PBS for 30 min, and then rinsed with ddH_2_O; then, they were stained with Alcian blue (pH 2.5, Muto Pure Chemicals, Tokyo, Japan) for 60 min. Afterwards, cells were washed with 3% acetic acid for three minutes to remove nonspecific staining and washing with PBS.

### 2.12. Masson’s Trichrome Staining

The sections were stained with Masson’s Trichrome staining by following the protocol of Masson’s Trichrome staining kit (Bio-Optica, Milano, Italy). The Zeiss Axiophot microscope (Carl Zeiss AG, Werk Göttingen, Germany) was used to observe illumination through a bright light, and photographs were acquired by a Discovery C30 camera (Tucsen Imaging Technology Co., Ltd. Fujian, China).

### 2.13. Immunofluorescence

The sections which were 5 μm thick were incubated in 0.1% trypsin (Gibco, ThermoFisher, Carlsbad, CA, USA) at 37 °C for 15 min for enzymatic antigen retrieval. The sections were then blocked with blocking buffer consisting of 5% milk in PBS, followed by incubation in a rabbit polyclonal antibody to collagen type II (diluted 1:200, Abcam, Cambridge, MA, USA) or a goat monoclonal antibody to aggrecan (diluted 1:200) overnight at 4 °C, and then incubation of a secondary antibody (diluted 1:200, Alexa Fluor 488 goat anti-rabbit, ThermoFisher, Carlsbad, CA, USA) for an hour at room temperature. Nuclei were stained with DAPI mounting gel (Vectashield, Vector Laboratories, Burlingame, CA, USA) to identify cellular nuclei. The images were taken with a Zeiss fluorescence microscope.

### 2.14. Magnetically Modulated Fluorescence (MMF)

Fluorescence spectra of FNDs suspended in aqueous solution were acquired by using a MMF spectrometer built in-house [34]. The spectrometer was equipped with a continuous-wave 532 nm laser (DPGL-2100F, Photop Suwtech, Shanghai, China), a dichroic beam splitter (Z532RDC, Chroma, Taoyuan, Taiwan), a long-working distance microscope objective (50Å~, NA 0.55, Mitutoyo, Kanagawa Prefecture, Japan), a long-pass edge filter (E550LP, Chroma, Taoyuan, Taiwan), and a multichannel analyzer (C7473, Hamamatsu, Japan). Backward fluorescence was collected through the same objective to avoid spectral distortion due to strong light-scattering by FNDs. To eliminate background noise, we magnetically modulated the FND fluorescence signal, and then applied fast Fourier Transform (FFT) to extract concentration information from the measured fluorescence intensities. This was achieved by applying a time-varying magnetic field of 20 mT from a round electromagnet (EM400-12-212, APW, Kuala Lumpur, Malaysia) to the sample solution held in a 10 mm cuvette with a frequency of 2 Hz. For every wavelength, we obtained a time-domain spectrum, with the magnetic field periodically switching on and off. A MATLAB program analyzed the time evolution of the spectra by performing FFT to yield intensities at the modulation frequency. The FND spectra were finally restored by plotting the demodulated fluorescence intensities against the wavelength. To conduct the analysis, the cells were first sonicated in a cuvette for 1 h to break up the cells. The amounts of the internalized FNDs in the cells were then determined from the fluorescence intensities against a calibration curve. To enable FND quantification in the xenografts by the MMF method, we digested the xenografts in aqua regia/H_2_O_2_ mixtures to release the nanoparticles into the solution. Fluorescence intensities were then measured directly for FNDs in the tissue digests without extraction or other separation procedures to avoid loss of the particles during centrifugation or filtration. An important thing to note is that aqua regia is a mixture of nitric acid and hydrochloric acid with an optimal molar ratio of 1:3. Regarding chemical safety, it is important to add the nitric acid to the hydrochloric acid during preparation. Since the acid mixture is unstable, it should be prepared in a small amount and used immediately.

### 2.15. Statistical Analysis

Results of multiple observations are presented as means ± S.E.M. For analysis of multivariate data, group differences were assessed using T tests, one-way or two-way ANOVA, followed by Turkey’s multiple comparison tests by using GraphPad Prism version 7.00 for Windows (GraphPad Software, La Jolla, CA, USA). All the experiment results in this research were repeated at least three times. *p* < 0.05 was recognized as the statistical significance between categories.

## 3. Results and Discussion

### 3.1. FNDs Preparation

**Figure 1:**
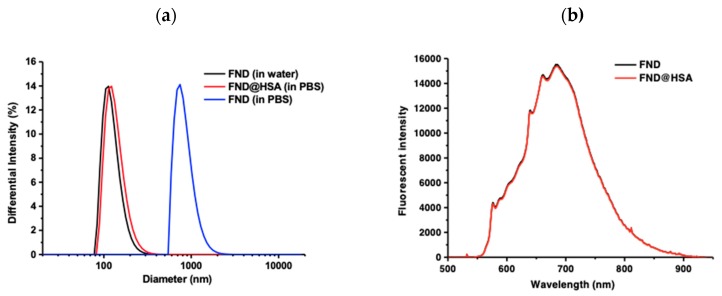
(**a**) Dynamic light scattering measurements of the size distributions of FNDs with or without HSA conjugation in distilled deionized water and PBS. (**b**) Fluorescence spectra of FNDs before and after conjugation with HSA (FND@HSA). The emission spectrum was acquired by laser excitation at 532 nm.

As shown in Figure 1a, the FNDs aggregated in PBS, which made FNDs unable to suspend homogenously in the culture medium. Therefore, FNDs were conjugated with HSA by sonicating the FNDs in distilled deionized water for 15 min. They were then mixed with the protein at a weight ratio of FND:HSA ≈ 1:1 by gentle shaking at room temperature for 2 h to allow physical adsorption. After removal of unbound HSA by centrifugation (15,000 rpm for 5 min), the precipitate was then washed with phosphate-buffered saline (PBS) [34]. As shown in Figure 1a, FNDs conjugated with HSA would prevent FNDs aggregation in PBS without interference of fluorescent spectra of FNDs (Figure 1b). FNDs, like other nanoparticles, are prone to precipitation in physiologic conditions, which makes them difficult to label the cells homogenously. As described in the Materials and Methods section, FNDs with carboxylated/oxidized surface functionalities under strong oxidative-acid treatment could have non-covalent interactions with serum albumin, a colloid-stabilization agent, to prevent agglomeration of FNDs. The FNDs were conjugated with serum albumin without desorption, as reported [36,37]. To test whether the HSA has been taken inside the hASCs, the protein lysates of hASCs after incubation with HSA alone or FND-conjugated HSA for 4 and 6 h were examined with SDS-PAGE and stained with Coomasie blue. The increase of HSA in the cell lysates of hASCs incubated with FND-conjugated HSA was noted, compared to hASCs incubated with HSA alone or without HSA (Figure S4). Taken together, these results show that conjugation of FNDs and HSA was stable and could be used for cell labeling.

### 3.2. Characterization of FND—Labeled hASCs

**Figure 2:**
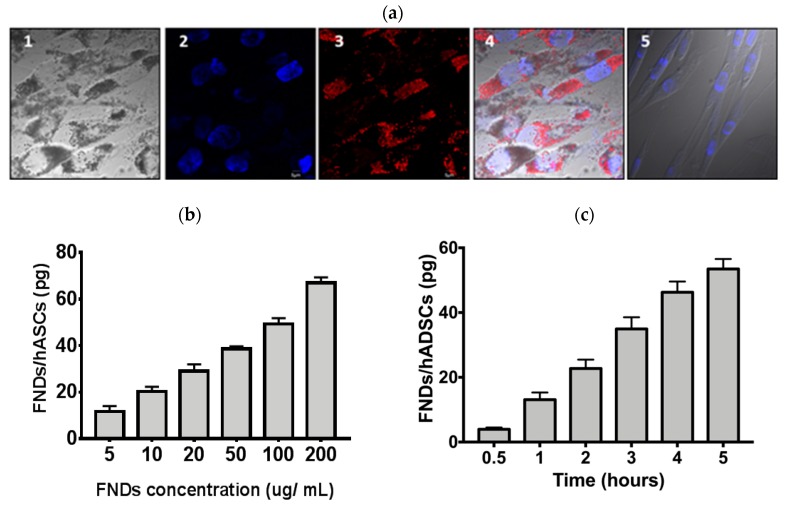
Characterization of fluorescent nanodiamond (FND)-labeled human adipose-derived stem/stromal cells (hASCs). (**a**) hASCs were incubated with 100 μg/mL for 4 h. The fluorescent images of FND-labeled hASCs were obtained by a fluorescent microscope. Cell nuclei were stained by 4′,6-diamidino-2-phenylindole (DAPI). The images were shown: (1) bright-field, (2) DAPI, (3) FNDs, (4) the merged image, and (5) hASCs without FNDs. Scale bar: 20 μm. (**b**) hASCs were incubated with FNDs at concentrations of 10–200 μg/mL for four hours. Quantification of FNDs in the cells was performed and analyzed by magnetically modulated fluorescence (MMF). (**c**) hASCs were incubated with FNDs in a time-course manner (half to five hours) at 100 μg/mL. The intracellular FNDs were subsequently analyzed by MMF.

To investigate the number of FNDs that could be loaded into the hASCs, the hASCs were incubated with FNDs at the indicated times and concentrations. As shown in Figure 2a, many red fluorescent signals were noted in the cytoplasm when hASCs were labeled with 100 μg/mL FNDs for 4 h incubation. The fluorescence of FNDs was taken by the excitation at 532 nm and collection of the emission at 685 nm, corresponding to the phonon sidebands of an electronic transition of NV^−^ centers. FNDs were probably taken into the cytoplasm via receptor-mediated endocytosis, emitting a distinct fluorescence which was detected with the microscope, as previously reported [32,33] to account for the engulfing FNDs, and which could be a possible hypothesis of the hASCs engulfing the FNDs. We further applied the background-free detection method to quantify the amount of FNDs taken up by cells. hASCs were incubated with FNDs at the particle concentration of 5–200 μg/mL, and the cellular uptake was subsequently analyzed by a home-built MMF spectrometer after removal of free FNDs in the medium [34] (Figure 2b). When the hASCs were incubated with 100 μg/mL FNDs at 37 °C for four hours, an average weight of 49.3 pg (or ~2.7 × 10^4^ particles) FNDs per cell were noted. As shown in Figure 2c, the amounts of FNDs taken up by hASCs in the six time points during the 30 min-to-5 h time frame could be determined by MMF, as described in the Materials and Methods section.

### 3.3. Proliferations and Differentiation of hASCs Not Affected by FNDs

**Figure 3:**
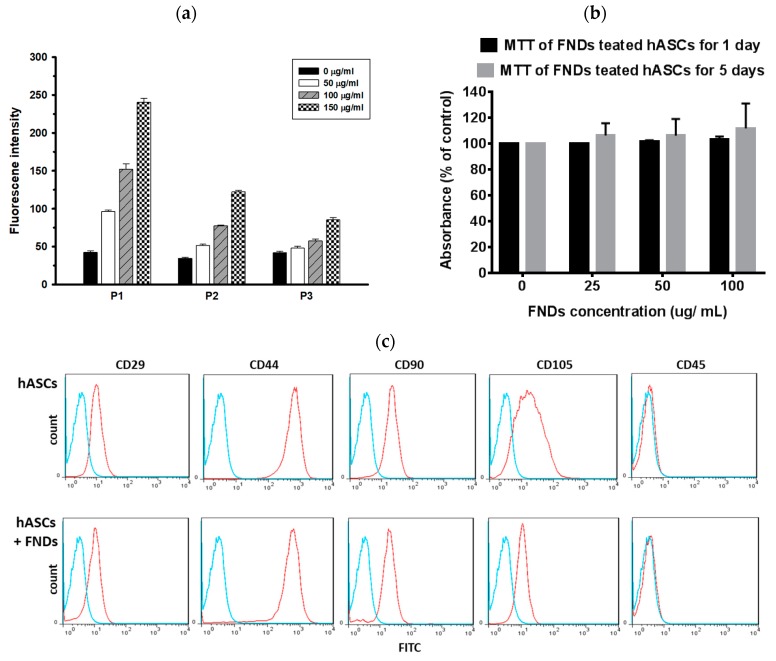
(**a**) Fluorescence intensity of FND-labeled hASCs was detected after each passage. 1 × 10^5^ hASCs were labeled with FNDs (0, 50, 100, 150 μg/mL). Values are means ± S.E.M. from three independent experiments. Data were assessed using one-way ANOVA, followed by Turkey’s multiple comparison tests. (**b**) 1 × 10^5^ hASCs were treated with FNDs (0, 25, 50, 100 μg/mL) for one and five days, followed by MTT analysis. Values represent the means ± S.D. of three independent experiments. Data were assessed using T tests. (**c**) Surface markers of hASCs. Representative flow cytometry analysis demonstrated positives for mesenchymal stem/stromal cells markers CD29, CD44, CD90, and CD105, but negative for CD45. Blue lines indicate the isotype control. FITC-A, fluorescein isothiocyanate area.

The intensity of fluorescent signals of FNDs increased in a dose-dependent manner, and the signals of FNDs in cells could still be detected with increasing passages of cells (Figure 3a). Since FNDs have been illustrated as cell-tracking materials [32,38,39], we next examined whether the FNDs showed cytotoxicity in hASCs. MTT analysis, a cell viability assay, was performed with 1 × 10^5^ hASCs labeled with FNDs in a dose-dependent manner with one- or five-day incubation. No significant difference was noted in each group in terms of cytotoxicity and cell proliferation (Figure 3b). Flow cytometry analyses were performed to compare the expression pattern of stem/stromal cell-associated specific surface markers of hASCs with or without FND labeling. Positive expression of CD29, CD44, CD90, and CD105 were observed in the hASCs regardless of the presence of FND, but was negative for CD45 (Figure 3c). It was illustrated that FND-labeled hASCs possessed consistent characteristic mesenchymal stem/stromal cell (MSC) phenotypes as hASCs.

To further evaluate the effect of FNDs on hASCs’ multilineage differentiation capability of hASCs, cells were pre-incubated with 100 μg/mL FNDs and cultured in an adipogenesis, osteogenesis, or chondrogenesis induction medium, respectively, for three weeks to allow cell differentiation. As shown in the Supplementary Figure S1a, FND-labeled hASCs displayed no difference in adipogenic differentiation compared to the control sample, as evaluated by Oil-Red O staining. The hASCs with FND labeling also showed no differences in osteogenesis compared to the control sample, as determined by Alizarin Red S staining (Supplement Figure S1b). Similar results were found in chondrogenic differentiation stained with Alcian Blue, which indicates that the FND-labeled hASCs maintained chondrogenic differentiation activity (Supplement Figure S1c). Overall, the FND-labeled hASCs maintained their potential for adipogenic, osteogenic, and chondrogenic differentiation.

### 3.4. hASCs Maintained the Volume of Xenografts Containing Decellularized Cartilage and Fibrin Glue Polymer

**Figure 4:**
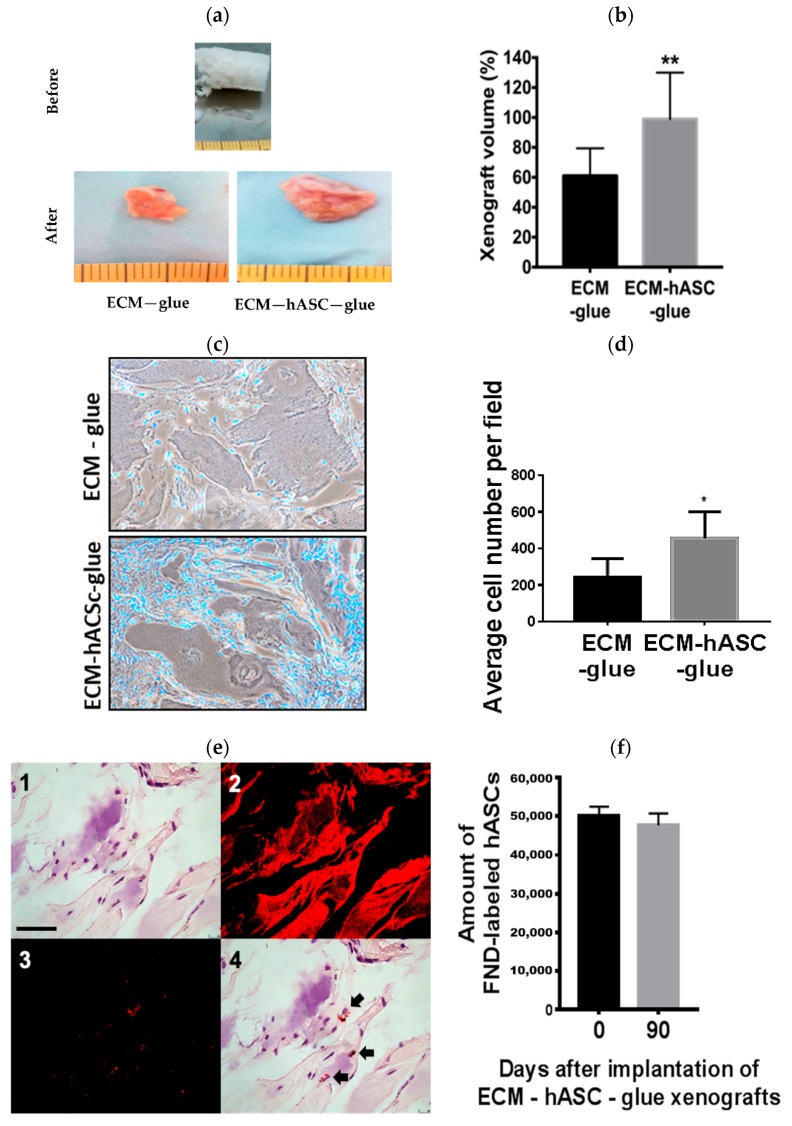
The xenografts composed of diced decellularized cartilage ECM and fibrin glue polymer with or without FND-labeled hASCs were transplanted subcutaneously into nude mice for three months. (**a**) Macroscopic appearance of xenografts containing ECM and fibrin glue polymer with or without FND-labeled hASCs before implantation (above) and three months afterwards (below). (**b**) Volume of the xenografts were measured. There was a statistic significant difference in the volume of xenograft between the groups of ECM-glue and ECM-hASC-glue. The values were means ± S.D. (n = 8). ** *p* < 0.01 versus control. Data were assessed using T tests for analysis. (**c**) Histological analysis of xenografts in both groups. Comparison of cell density by DAPI between groups of ECM-glue and ECM-hASC-glue three months afterwards (Original magnification × 400). (**d**) Cell numbers were quantified under microscope per field, as described in the Materials and Methods section, by Image J software. The ECM-hASC-glue group has significantly increased cell numbers compared to the ECM-glue group. The values were mean ± S.E.M. (n = 8). ** *p* < 0.01 versus control. * *p* < 0.05 versus control. Data were assessed using T tests for analysis. (**e**) Identification of FND-labeled hASCs in a xenograft tissue section by confocal microscopy: (1) H&E staining image, (2) confocal fluorescent image without time gating, (3) time-gated confocal fluorescent image, and (4) merged image of (1) and (3). Black arrows denote FND-labeled hASCs. Scale bar: 50 μm. (**f**) Quantitative detection of FND-labeled hASCs with MMF. The corresponding percentages of FND-labeled hASCs found in the implanted xenografts 90 days later. The values were means ± S.E.M. (n = 3). Data were assessed using T tests for analysis.

Three groups of xenografts were performed in an in vivo experiment, including ECM-glue, ECM-hASC-glue, and hASC-glue, as described in the Materials and Methods section. The combination of fibrin glue polymer and diced decellularized cartilage ECM served as an appropriate 3D scaffold with adhesive properties maintaining the xenograft constructs [23]. Furthermore, fibrin is applied extensively for clinical applications because of its clinical safety [27]. To test whether the fibrin scaffolds containing diced decellularized cartilage ECM could make in vivo chondrogenesis of hASCs without in vitro induction, xenografts were implanted into the subcutaneous layer of nude mice for three months (Figure 4a above). The diameter of xenografts post three month-implantation from the ECM-hASC-glue group was approximately 12 mm (Figure 4a, bottom right), whereas the diameter of xenografts post three-month implantation from the ECM-glue group was approximately 6 mm (Figure 4a bottom left). Three months afterwards, the volume of the xenografts from the ECM-glue and ECM-hASC-glue groups were 64.09% ± 15.8 and 98.97% ± 17.35 compared to the original volume of xenografts, respectively (Figure 4b). As shown in Supplementary Figure S2, the appearance on the skin of the hASC-glue group was flat, as indicated by the arrows one month after implantation. To further examine whether the hASCs were associated with maintenance of the volume of xenografts, histological analysis of the xenografts was performed. As shown in Figure 4c,d, the increased number of cell nuclei stained by DAPI (blue dots) was noted in the group of ECM-hASC-glue compared to ECM-glue, indicating that hASCs could significantly maintain the xenograft volume in vivo.

The combination of the diced irradiated cartilages and fibrin glues has been used in the clinical treatment in recent years. One of the disadvantages of the current method is the reabsorption of the graft with decreased volume, which made the clinical outcome unsatisfactory. Therefore, stem-cell-based therapies combined with scaffolds could be one of the promising strategies in chondrogenesis. The grounded ECM is a loose, pulverized material; hence, the xenograft constructs made of ECM-alone and ECM-hASCs could not be maintained without fibrin glue polymer in this study. Fibrin glue polymers used in this study could be maintained for 5 days, which might contribute to the formation of the xenograft construct in our experiment [28]. If we include glue-alone or hASCs-alone in the experimental group in our three-month experimental design, the volume of the xenografts could not be maintained for three months. The xenograft constructs of hASC-glue which disappeared one month after implantation might tell us the importance of ECM in volume maintenance. Taken together, the xenograft constructs could be supported by the decellularized ECM, and the volume of the xenograft could be maintained by hASCs. These findings are important for clinical applications, as they provide valuable information for understanding the mechanism of supportive volume in cartilage repair.

### 3.5. Long-Term Detection of FND-Labeled hASCs with an In Vivo Chondrogenesis Model

To further identify whether the implanted FND-labeled hASCs could be detected in the xenografts, we tracked the hASCs using the unique property of FNDs. FNDs contain the fluorophores NV^−^ centers with a fluorescence lifetime (τ) longer than 15 ns, which is substantially longer than that (τ ≈ 1–4 ns) of the endogenous and exogenous fluorophores commonly used in cell biology [40]. Deparaffinized samples with H&E staining were used for FNDs imaging using time-gated confocal fluorescence microscopy to achieve background-free detection [34]. As shown in Figure 4e, several cell nuclei were noted around the surface of decellularized ECM (Figure 4e(1)). The signals of FND-labeled hASCs were interfered by the strong background fluorescence and autofluorescence under a confocal microscope (Figure 4e(2)). The signals of FNDs could be clearly detected using time-gated confocal fluorescence microscopy (Figure 4e(3)). As shown in Figure 4e(4), the merged images clearly showed the location of FND-labeled hASCs in the H&E staining. Taken together, these findings indicated that FND-labeled hASCs could be detected in the xenograft without interference of background fluorescence three months afterwards. As shown in Figure 4f, numbers of implanted FND-labeled hASCs in the xenografts can be quantified by MMF, as described in the Materials and Methods section. To our knowledge, our study is probably the first experiment to detect FND-labeled cells for three months in an animal model.

On the strength of the chemical robustness of the nanomaterial, the unique magneto-optical properties of FNDs were still preserved and their fluorescence signals could be readily recovered from the acid digests by magnetic modulation at the concentration as low as 1 ug/mL. The recovery rate, determined by spiking a known amount of FNDs into the mouse tissue followed by digestion through the same process, was more than 90% [34]. Such quantitative analysis is inaccessible with molecular fluorophores such as organic dyes and fluorescent proteins because of the lack of chemical stabilities in strong acids.

### 3.6. Functional In Vivo Chondrogenesis

**Figure 5:**
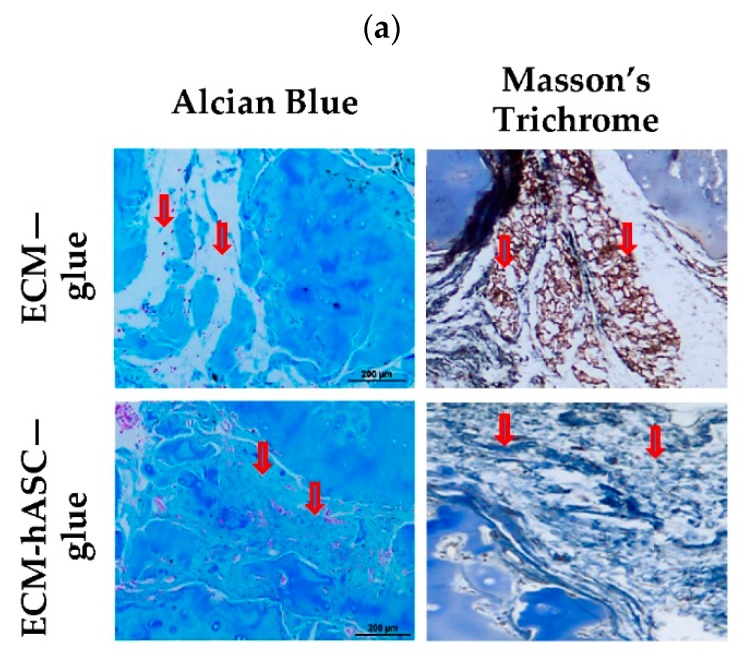

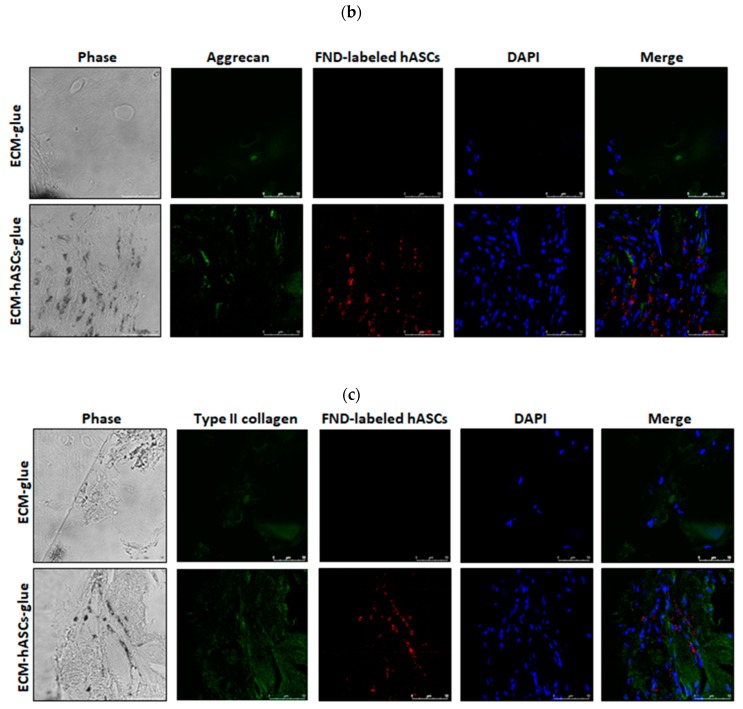
Functional in vivo chondrogenesis of hASCs. (**a**) The presence of glycosaminoglycans and collagen demonstrated chondrogenic differentiation by Alcian Blue and Masson’s Thrichrome staining, separately. Alcian Blue, Nuclear Fast Red, and Masson’s Thrichrome staining of the xenografts from ECM-hASCs-glue and ECM-glue groups three months afterwards. Upper panel represents the ECM-glue group. Lower panel represents the ECM-hASCs-glue group. Masson’s Trichrome staining of the ECM-hASCs-glue and ECM-glue groups after three months implantation (Original magnification × 200). (Arrows indicate the interstitial spaces.) (**b**) Immunofluorescent staining for Aggrecan for identifying glycosaminoglycan (green) and FND-labeled hASCs (red) in ECM-hASCs-glue and ECM-glue groups. (**c**) Fluorescence imaging of type II collagen (green) were presented in ECM-hASCs-glue and ECM-glue groups. Scale bar: 50 μm.

All specimens were sent for histological analysis and treated with Alcian Blue and Masson’s Thrichrome staining for detection of glycosaminoglycans and collagen, respectively. As shown in Figure 5a, Alcian Blue staining showed increased blue staining and red dots (cell nuclei) in the interstitial space in ECM-hASC-glue xenografts compared to the ECM-glue xenografts. Increased collagen (blue color) was also noted in the Masson’s Trichrome staining. Moreover, the results with Alcian Blue and Nuclear Fast Red (red color) staining showed higher cellularity in ECM-hASC-glue group compared to the ECM-glue group. This result is compatible with our previous results, as shown in Figure 4c. These findings indicated that increased glycosaminoglycans and collagen might be secreted by the chondrocyte-like cells derived from the hASCs. To further validate whether the FND-labeled hASCs underwent chondrogenic differentiation, the immunofluorescence stainings of the xenografts were performed for aggrecan and collagen II with the confocal microscope. As shown in Figure 5b, colocalization of FNDs-labeled hASCs (red color) and aggrecan (green color) was noted. Similar results were noted in collagen II staining in Figure 5c. The colocalization of signals of FNDs and chondrocyte-markers, including aggrecan and collage type II, might indicate in vivo chondrogenesis of hASCs. Taken together, functional in vivo chondrogenesis of the FND-labeled hASCs in xenografts was found, and these xenografts contain decellularized ECM and fibrin glue polymer three months afterwards.

## 4. Conclusions

This study demonstrates that the volume of xenograft constructs could be better maintained with increased cellularity in the ECM-hASCs-glue group compared to the ECM-glue group. In addition, increased cartilage proteins were noted in the ECM-hASCs-glue group by Masson’s trichrome and Alcian blue staining. Signals of chondrogenic markers, such as aggrecan and collagen type II colocalized with FND signals, might indicate in vivo chondrogenesis of hASCs in xenografts containing decellularized cartilage ECM and fibrin glue polymers. Furthermore, we introduced a FND-based method combining cell-detecting technology with quantitative tracking of transplanted cells in vivo, which may warrant future stem/stromal cell research into translational research.

## Supplementary Materials

The following are available online at https://www.mdpi.com/2073-4360/11/9/1391/s1, Figure S1: FNDs result in no alteration on cell differentiation derived from hASCs. Figure S2: Appearance of hASC-glue group nude mice before (left) or after one-month (right) implantation. Figure S3: ECM preparation.
