# Supplementary material for: Fluorescent Nanodiamonds Enable Long-Term Detection of Human Adipose-Derived Stem/Stromal Cells in an In Vivo Chondrogenesis Model Using Decellularized Extracellular Matrices and Fibrin Glue Polymer
Source: Polymers (Basel). 2019 Aug 23;11(9):1391. doi: 10.3390/polym11091391 (PMC6780225; doi:10.3390/polym11091391)

Supplement 1

Figure S1. FNDs result in no alteration on cell differentiation derived from hASCs.

$1X10^5$ hASCs were pre-incubated with 100 g/mL FNDs overnight and replaced with induction and culture medium of (**A**) adipogenesis, (**B**) osteogenesis or (**C**) chondrogenesis for 3 weeks. Differentiations were confirmed by Oil-Red O, Alizarin Red S and Alcian Blue staining respectively. (Magnification ×200)

Figure S1.

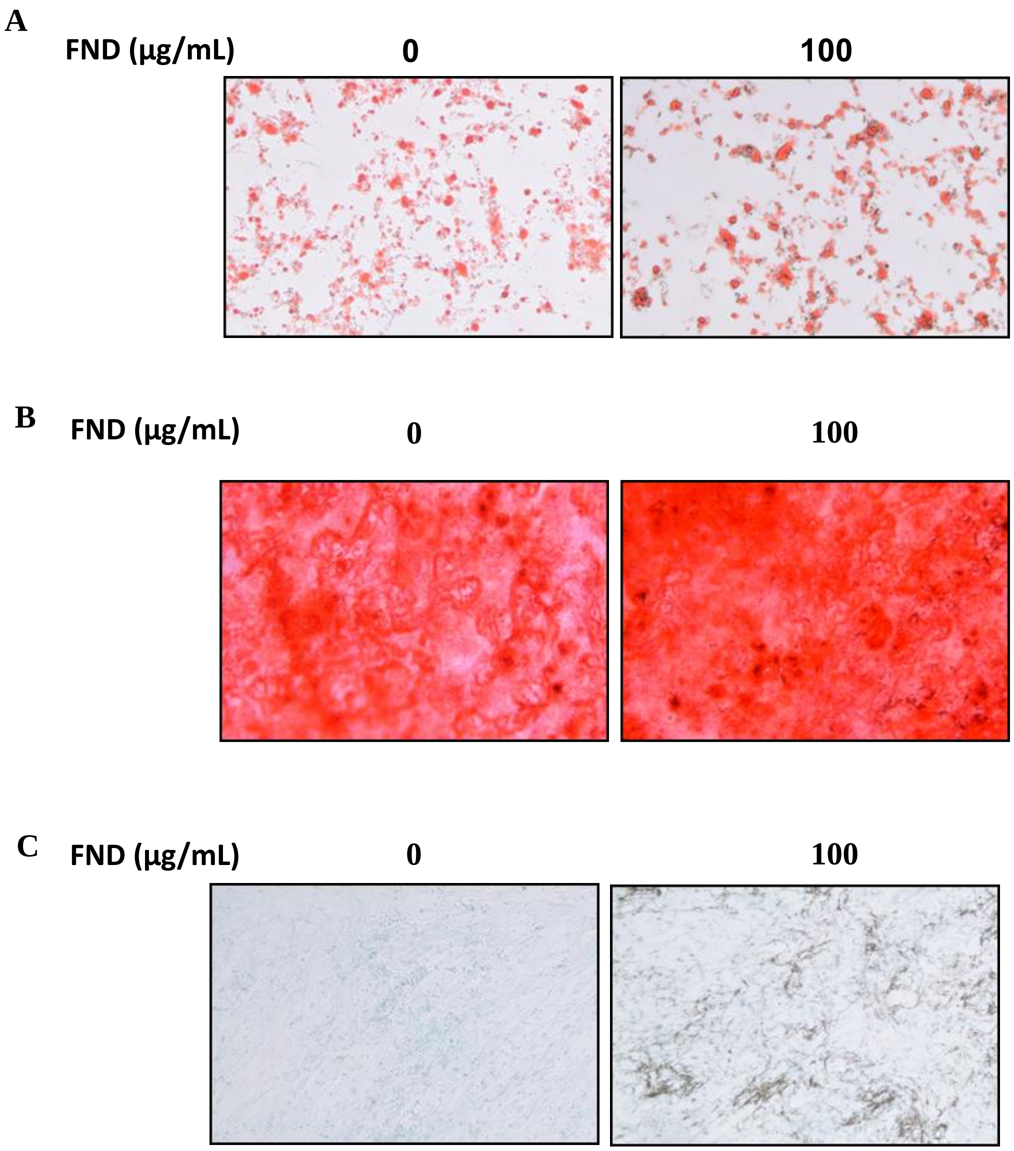

Supplement 2

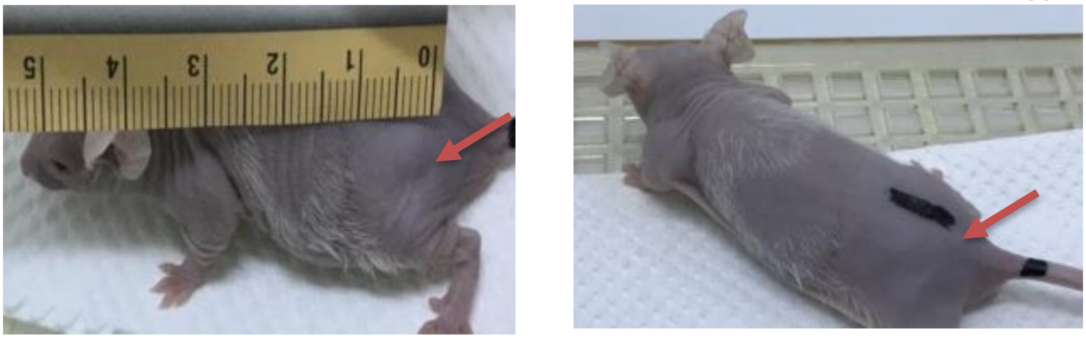

Figure S2.

Appearance of hASC – glue group nude mice before (left) or after one-month (right) implantation. The arrows indicate the locations of the implanted xenograft.

Supplement 3

Figure S3. (a) The allogenic irradiated costal cartilages were grounded into particles approximately 0.5 mm x 0.5 mm x 0.5 mm in size as decellularized cartilage ECM. (b) The ECM particles were then loaded into the syringe.

(a). **(b)**

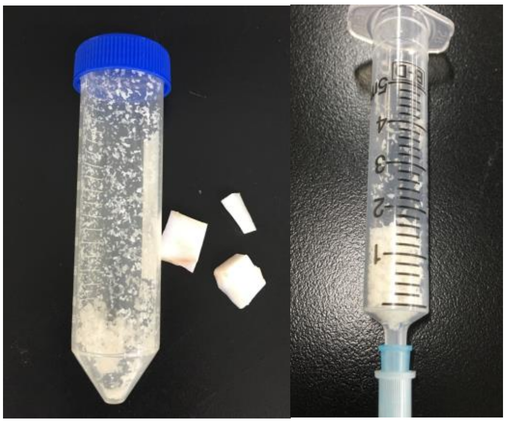

Supplement 4

Figure S4. SDS-PAGE analysis of HSA in hASCs incubated with HSA and FND@HSA. The hASCs were incubated, respectively with HSA, and 200 μg/mL FND conjugated HSA for 4 and 6 hours. The protein lysates of these hASCs after incubation or without incubation were subjected to SDS- PAGE analysis with Coomassie staining. The presence of HSA (red arrows) increased in the cell lysates of hASCs with FND conjugated HSA incubation for 4 and 6 hours compared with hASCs with HSA incubation. HSA served as a positive control.

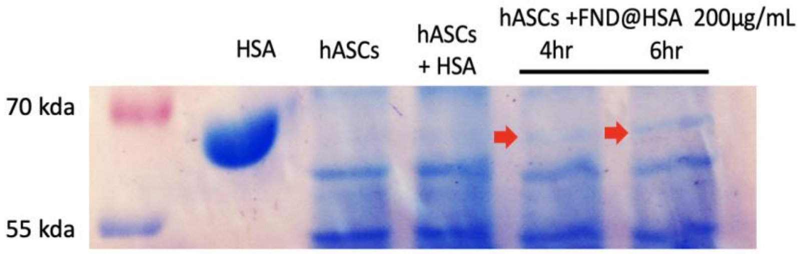

Supplement: Supplementary file 1 [file polymers-11-01391-s001.pdf]
